# Biological Landscape of Triple Negative Breast Cancers Expressing CTLA-4

**DOI:** 10.3389/fonc.2020.01206

**Published:** 2020-08-05

**Authors:** María G. C. Navarrete-Bernal, Mayte G. Cervantes-Badillo, Jose Fabián Martínez-Herrera, César O. Lara-Torres, Raquel Gerson-Cwilich, Alejandro Zentella-Dehesa, María de Jesús Ibarra-Sánchez, José Esparza-López, Juan J. Montesinos, Víctor Adrián Cortés-Morales, Diego Osorio-Pérez, Diana A. Villegas-Osorno, Eduardo Reyes-Sánchez, Pablo Salazar-Sojo, Luis F. Tallabs-Utrilla, Sandra Romero-Córdoba, Leticia Rocha-Zavaleta

**Affiliations:** ^1^Departamento de Biología Molecular y Biotecnología, Instituto de Investigaciones Biomédicas, UNAM, Ciudad de Mexico, Mexico; ^2^Departamento de Medicina Genómica y Toxicología Ambiental, Instituto de Investigaciones Biomédicas, UNAM, Ciudad de Mexico, Mexico; ^3^Programa Institucional de Cáncer de Mama, Instituto de Investigaciones Biomédicas, UNAM, Ciudad de Mexico, Mexico; ^4^American British Cowdray Medical Center, Cancer Center, Ciudad de Mexico, Mexico; ^5^American British Cowdray Medical Center, Pathology Service, Ciudad de Mexico, Mexico; ^6^Biochemistry Department, Instituto Nacional de Ciencias Médicas y Nutrición Salvador Zubirán, Ciudad de Mexico, Mexico; ^7^Red de Apoyo a la Investigación (RAI), Universidad Nacional Autónoma de Mexico-Instituto Nacional de Ciencias Médicas y Nutrición Salvador Zubirán, Mexico City, Mexico; ^8^Laboratorio de Células Troncales Mesenquimales, Unidad de Investigación Médica en Enfermedades Oncológicas, Hospital de Oncología, Centro Médico Nacional Siglo XXI, Instituto Mexicano del Seguro Social (IMSS), Ciudad de Mexico, Mexico; ^9^Escuela de Medicina, Universidad Panamericana, Ciudad de Mexico, Mexico

**Keywords:** triple-negative breast cancer, CTLA-4, immunotherapy, immune landscape, cancer genomics

## Abstract

Patients with triple-negative breast cancer (TNBC) have a poor prognosis, partly because of the absence of targeted therapies. Recognition of the key role of immune responses against cancer has allowed the advent of immunotherapy, focused on the inhibition of negative immune checkpoints, such as CTLA-4. CTLA-4 is also expressed in some cancer cells, but its activity in tumor cells is not completely understood. Thus, the aim of the present work was to determine the biological landscape and functions of CTLA-4 expressed in TNBC cells through preclinical and *in silico* analysis. Exploration of CTLA-4 by immunohistochemistry in 50 TNBC tumors revealed membrane and cytoplasmic expression at different intensities. Preclinical experiments, using TNBC cell lines, showed that stimulation of CTLA-4 with CD80 enhances activation of the ERK1/2 signaling pathway, while CTLA-4 blockade by Ipilimumab induces the activation of AKT and reduces cell proliferation *in vitro*. We then developed an analytic pipeline to define the effects of CTLA-4 in available public data that allowed us to identify four distinct tumor clusters associated with CTLA-4 activation, which are characterized by enrichment of distinctive pathways associated with cell adhesion, MAPK signaling, TGF-ß, VEGF, TNF-α, drug metabolism, ion and amino acid transport, and KRAS signaling, among others. In addition, blockade of CTLA-4 induced increased secretion of IL-2 by tumor cells, suggesting that the receptor regulates cellular functions that may impact the immune microenvironment. This is relevant because a deep characterization of immune infiltrate, conducted using public data to estimate the abundancies of immune-cell types, showed that CTLA-4-activated-like tumors present a conditional immune state similar to an escape phenotype exploited by cancer cells. Finally, by interrogating transcriptional predictors of immunotherapy response, we defined that CTLA-4 activation correlates with high immune scores related to good clinical predicted responses to anti-CTLA-4 therapy. This work sheds new light on the roles of activated CLTA-4 in the tumor compartment and suggests an important interplay between tumor CLTA-4-activated portraits and immune-infiltrating cell populations.

## Introduction

Breast cancer is the most prevalent invasive malignancy, and the most common cause of cancer-related death in women worldwide (1). Triple-negative breast cancer (TNBC) is a subtype that lacks the expression of estrogen receptor (ER), progesterone receptor (PgR), and human epidermal growth factor receptor 2 (HER2). Overall, TNBC represents 10–20% of all breast cancer cases (2), being more prevalent in women of Hispanic (3) and African descent (4). TNBC tumors exhibit an unfavorable clinical evolution, given the lack of specific targeted therapies (5). Currently, the standard treatment for TNBC patients is based on chemotherapy (6), and although the initial clinical response is acceptable, the overall prognosis for these patients is poor, compared with other breast cancer subtypes. Thus, there is a great need for the development of new therapeutic options for this pathology.

Recently, the recognition of the key role of immune responses against cancer cells has allowed the advent of immunotherapy based on immune checkpoint inhibitors. Immune checkpoints are negative regulators of cytotoxic T-lymphocyte (CTL) activity. CTLs are the main effector cells that recognize tumor antigens and mediate immune anti-tumor responses; CTL activity is regulated by the interplay between co-stimulatory pathways and checkpoints. After tumor antigen presentation, CTL-associated CD28 receptor binds to B7 (CD80/CD86) on the surface of antigen-presenting cells (APCs), resulting in CTL activation. Afterwards, the cytotoxic T- lymphocyte-associated antigen 4 (CTLA-4) is translocated to the CTL membrane, where it competes for and binds to B7, with a higher affinity than CD28, inhibiting the previously activated pathways (7). CTLA-4 upregulation in cancer patients is recognized as an important mediator of immune evasion. Thus, efforts to block CTLA-4 resulted in the advent of the monoclonal anti-CTLA-4 antibody, Ipilimumab, which was approved to be used as a checkpoint inhibitor for the treatment of melanoma (8, 9). However, the proportion of patients who respond to Ipilimumab remains modest (~22% objective response rate), as tumors have multiple mechanisms of immune evasion (10).

The use of anti-CTLA-4 immunotherapy in other types of cancer has been hampered by the limited understanding of driving responses and resistance pathways. For instance, breast tumors have been considered immunologically quiescent or “cold” tumors. However, recent multi-omic profiling has provided robust characterization of the immune portrait of breast cancers, describing TNBC as a dynamic and heterogeneous immunogenic subtype (11, 12). Since immunotherapy aims at stimulating and restoring anti-tumor immune responses, it is reasonable to hypothesize that the immune microenvironment surrounding tumors is crucial for its success. Recent reports that integrate transcriptional-based deconvolution algorithms and effector-regulatory immune signatures in TNBC demonstrated the existence of three immunoclusters. Among them, an immune-activated cluster was shown to be associated with an inflammatory phenotype, high T and natural killer (NK) cell infiltration, and a favorable prognosis. Interestingly, this immunocluster was also associated with enriched expression of CTLA-4 (13). The expression of CTLA-4 in various tumor types and cancer cell lines has been previously described (14–17). It has also been reported that expression of CTLA-4 occurs in around 50% of breast carcinomas, but not in normal breast tissues (18). However, evidence describing CTLA-4 signal transduction pathways in TNBC cells, and the potential effects of its modulation, is still limited.

Thus, here we characterized the biological role of CTLA-4 expression in TNBC cells through preclinical *in vitro* experiments and benchmark analysis. Immunohistochemistry analyses with a robust scoring system of TNBC biopsies corroborated CTLA-4 expression in different cellular compartments. We then investigated CTLA-4 functions and associated signaling pathways by activating or blocking the receptor on TNBC cell lines. On the basis of public gene expression profiles of TNBC, the transcriptional landscape of tumors over-expressing CTLA-4 with activated downstream pathways was described. Additionally, we characterized the interactions between tumor-expressed CTLA-4 and immune infiltration. Finally, an overview of the possible clinical immunotherapy responses of tumors with activated CTLA-4-associated signaling was explored through public signatures. Improving our knowledge on the activity of CTLA-4 on tumor cells will help to understand the potential effects of the receptor on the clinical response to immunotherapy.

## Materials and Methods

### Clinical Samples and Cell Lines

A total of 50 patients diagnosed with invasive TNBC between 2005 and 2019, at the American British Cowdray Medical Center (ABC Medical Center) (Mexico City, Mexico) were recruited. The study was approved by the institutional research and ethics committees from the ABC Medical Center. Patients were selected if: (i) they were females; (ii) had histological diagnosis, (iii) had molecular diagnosis showing negative ER, PgR, and HER2; (iv) had electronic or physical clinical record to obtain clinical information on the stage of diagnosis and treatment; and (v) had a tumor percentage >10%. Paraffin-embedded tissue sections were retrospectively collected, and immunochemistry information, including ER, PgR, and HER2 expression, and Ki67 index were collected from the Pathology Department of the ABC Medical Center. The clinicopathological characteristics of the study population are summarized in Table 1.

**Table 1:** Clinicopathological characteristics of TNBC cases.

Age	Mean (SD)	54.9	(15.9)
Smoking	Yes	20	(40.0 %)
	No	30	(60.0%)
Body mass index	Mean (SD)	26.6	(5.3)
Cancer family history	Breast	13	(26.0%)
	Pancreas	8	(16.0%)
	Gastrointestinal	3	(6.0%)
	Cervix	3	(6.0%)
	Prostate	2	(4.0%)
	Kidney	2	(4.0%)
	Lung	2	(4.0%)
	Sarcoma	2	(4.0%)
	Melanoma	2	(4.0%)
	CNS glioma	2	(4.0%)
	Leukemia	2	(4.0%)
	Colon	1	(2.0%)
	Lymphoma	1	(2.0%)
Stage	Early	17	(34.0%)
	Locally advanced	24	(48.0%)
	Metastatic	7	(14.0%)
	NA	1	(4.0%)
Tumor biopsy	Breast	42	(84.0%)
	Metastasis	7	(14.0%)
	NA	1	(2.0%)
Chemotherapy	Neoadyuvant	26	(52.0%)
	Non-neoadyuvant	23	(46.0%)
	NA	1	(2.0%)
Follow Up	Alive	15	(30.0%)
	Deceased	6	(12.0%)
	Lost to follow up	29	(29.0%)
CTLA-4 positivity	Lymphocytes^*^	45	(90.0%)
	Tumor cells^**^	35	(70.0%)
CTLA-4 score	TC0	15	(30.0%)
	TC1	7	(14.0%)
	TC2	21	(42.0%)
	TC3	7	(14.0%)

* *Samples showing a density of tumor-infiltrating lymphocytes >1%*.

** *Considering membrane staining exclusively. NA, Not available information*.

TNBC cell lines MDA-MB-231 (ATCC® HTB-26™), HCC1937 (ATCC® CRL-2336™), DU4475 (ATCC® HTB-123™), and HCC70 (ATCC® CRL-2315™) were purchased from the American Type Culture Collection (ATCC, Rockville, MD, USA). All cell lines were maintained in Roswell Park Memorial Institute (RPMI)-1640 medium (Biowest SAS, Nuaillé, France) supplemented with 10% fetal bovine serum (FBS; Biowest SAS, Nuaillé, France), 100 mg/ml of streptomycin, and 100 U/ml of penicillin (Invitrogen, Carlsbad, CA, USA).

### Immunohistochemistry (IHC) and CTLA-4 Evaluation

CTLA-4 expression was evaluated by IHC in the selected TNBC biopsies. 2-μm sections from formalin-fixed paraffin-embedded tissue were deparaffinized and rehydrated. Antigen retrieval and immunodetection were achieved by using the Mouse/Rabbit Immuno-Detector DAB HRP Brown Detection System (Bio SB, Inc. CA, USA). Tissue sections were stained with anti-CTLA-4 (Cat. F-8 sc-376016, Santa Cruz Biotechnology, Inc. TX, USA) diluted 1:30. Standard hematoxylin-eosin (H-E) staining was performed for each tumor sample and CTLA-4 staining intensity was analyzed by an expert pathologist. Assessment of CTLA-4 expression on tumor cells was defined based on the criteria previously published for scoring PD-L1 (19). This protocol defines a positive stain as the percentage of membrane staining of any intensity in tumor cells, and tumor cells are scored as the proportion of tumor area. The scores were categorized as follows: T0 (negative, <1%), T1 (low/weak, >1 and <5%), T2 (medium, >5 and <50%) and T3 (high/strong, >50%) (Roche, VENTANA PD-L1). The slides were scanned using an iScan Coreo Au (Ventana Medical Systems, Inc, AZ, USA).

### Reverse Transcriptase PCR Analysis

Total RNA was isolated from the cell lines using the RNeasy Mini Kit (Qiagen, Valencia, CA, USA) following the manufacturer's protocol. Reverse transcriptase-polymerase chain reaction (RT-PCR) for the detection of CTLA-4 transcripts was carried out using the SuperScript One-Step RT-PCR System (Qiagen, Valencia, CA, USA), with the following oligonucleotides: 5′-ATGGCTTGCCTTGGATTTCAGCGGCACAAGG-3′ and 5′-TCAATTGATGGGAATAAAATAAGGCTGAAATTGC-3′, which amplify a 660-bp fragment corresponding to the entire CTLA-4 coding sequence (20). The reverse transcription reaction was carried out at 50°C for 30 min. The CTLA-4 DNA amplification was performed using the following PCR program: 95°C for 15 min, followed by 40 cycles of 94°C for 1 min, 55°C for 1 min, 72°C for 1 min, and a final extension at 72°C for 10 min. Amplification of the β-actin gene, using the forward 5′-GGG TCA GAA GGA TTC CTA TG-3′ and reverse 5′-GGT CTC AAA CAT GAT CTG GG-3′ oligonucleotides, was included as an internal control. RNA from phytohemagglutinin-L-activated human peripheral blood mononuclear cells (PBMC) was included as a positive control.

### Flow Cytometry Analysis

The expression of cytoplasmic CTLA-4 was analyzed by flow cytometry using the APC-conjugated mouse monoclonal anti-human CD152 antibody (Clone BNI3; BD Biosciences. San Jose, CA, USA). Cell surface CTLA-4 was evaluated by flow cytometry using mouse monoclonal Fluorochrome-labeled anti-CTLA-4 (Cat: 369612; Biolegend San Diego, CA, USA). CD80 was analyzed using the Phycoerythrin-conjugated anti-B7-1/CD80 (Clone No. 3771; R&D Systems Inc. Minneapolis, MN, USA). CD86 was evaluated using the APC-conjugated anti-B7-2/CD86 (Clone No. 37301; R&D Systems Inc. Minneapolis, MN, USA). Phytohemagglutinin-activated PBMC were included as a positive control for the expression of CTLA-4. Human peripheral blood monocytes cultured *in vitro* were included as a positive control for the expression of CD80 and CD86 (21). The cells were analyzed in a FACS Canto II Flow Cytometer (BD Biosciences Co. San Jose, CA, USA), capturing 10,000 events per sample. The percentages of positive cells and average fluorescence intensities were obtained and analyzed with the FlowJo 10 software.

### Cell Proliferation and Invasion Assays

To evaluate cell proliferation, cells were seeded in 96-well plates at a density of 10,000 cells per well and incubated with either recombinant human CD80 (0.025, 0.15, and 1 μg/ml; Abcam, Cambridge, MA, USA) or Ipilimumab (1, 5, and 10 μg/ml; Bristol-Myers Squibb Company). Cell viability was measured at 24, 48, and 72 h after incubation using the colorimetric 3-(4,5-dimethylthiazol-2-yl)-2,5-diphenyltetrazolium bromide (MTT) assay.

To assess cell invasion, a transwell assay using extracellular matrix-coated Boyden chambers (Sigma-Aldrich, St. Louis, MO, USA) was performed. Cells were seeded in the upper chamber in FBS-free medium and treated with Ipilimumab (10 μg/ml) or CD80 (1 μg/ml) for 24 h. FBS-supplemented medium was added to the lower chamber. Cells which had passed through the matrix-coated membrane were recovered from the lower compartment, stained with the CellTracker Red reagent (Thermo Fisher Scientific Inc., Waltham, MA, USA) and evaluated in a Synergy H4 hybrid plate reader (BioTek Instruments Inc., Winooski, VT, USA) using the Gen5 software.

### Western Blot

For protein extraction, cells were resuspended in lysis buffer (50 mM Tris–HCl, pH 7.4; 150 mM NaCl; 1 mM EDTA; 1% NP40; 0.25% sodium deoxycholate), containing 100 μl/ml of protease inhibitor cocktail (Roche, Manheim, Germany) and 10 μl/ml of phosphatase inhibitors (Sigma-Aldrich, St. Louis, MO, USA). Protein concentration was quantified using the DC protein assay kit (BioRad Laboratories, Hercules, CA, USA). Proteins (30 μg) were resolved by 10% SDS-PAGE and transferred onto polyvinylidene fluoride (PVDF) membranes (Millipore, Billerica, MA, USA). The following antibodies were used for the detection of CTLA-4-associated signaling pathways: Phospho-p44/42 MAPK (ERK1/2) (Thr202/Tyr204) (Cat. 9101), phospho-Akt (Thr308) (244F9) (Cat. 4056), phospho-Akt (Ser473) (Cat. 9271), Akt (pan) (C67E7) (Cat. 4691), Snail (C15D3) (Cat. 3879), ZEB1 (D80D3) (Cat.3396), and PTEN (138G6) (Cat. 9559), all from Cell Signaling (Cell Signaling Technology, Inc., Danvers, MA, USA); ERK1 and ERK2 (ERK1/2) (Cat. GTX17942), phospho-ERK1/2 (Thr185 + Thr202 + Tyr204 + Tyr187 (Cat. GTX24819); and anti-GAPDH (Cat. GTX100118), Vimentin (Cat. GTX-100619), and Twist (GTX-127310), all from GeneTex (GeneTex Inc., Irvine, CA, USA); E-cadherin (Cat. sc-71009), Vinculin (Cat. sc-25336), β-catenin (Cat. sc-7963), Actin (Cat. sc-47778), all from Santa Cruz (Santa Cruz Biotechnology, Inc., Dallas TX, USA). Densitometric analysis was performed with ImageJ software.

### IL-2 Quantitative ELISA

Cells were incubated with Ipilimumab (10 μg/ml), CD80 (1 μg/ml), or left untreated for 48 h. Conditioned medium was harvested. Soluble IL-2 was determined using the Human IL-2 Mini TMB ELISA Development kit (PeproTech, Inc. NJ, USA) according to the manufacturer's protocol. Absorbance values were measured at 450 nm in a Synergy H4 hybrid plate reader (BioTek Instruments Inc. Winooski, VT, USA).

### Co-culture of CTLA-4-Expressing TNBC Cells and Peripheral Blood Mononuclear Cells

Blood samples were obtained from healthy female donors. Peripheral blood mononuclear cells (PBMC) were isolated by standard Histopaque density centrifugation (Histopaque-1077. Sigma-Aldrich, St. Louis, MO, USA). PBMC were cultured in RPMI-1640 medium supplemented with 10% FBS, 100 mg/ml of streptomycin and 100 U/ml of penicillin, and 2 mM L-glutamine (Invitrogen, Carlsbad, CA, USA). To induce the expression of CTLA-4, PBMC were stimulated with 7.5 μg/ml Phytohemagglutinin (PHA-P. Sigma-Aldrich, St Louis, MO, USA) in supplemented RPMI for 6 days. CTLA-4 expression was evaluated by flow cytometry. Stimulated PBMC (16 × 10^4^) were washed with PBS and co-cultured with HCC1937 or MDA-MB-231 (4 × 10^4^) in 96-well plates for 48 h. The effect of CTLA-4 blockade was evaluated by adding Ipilimumab (10 μg/ml). At the end of the experiment, non-adherent cells were harvested and the number of viable cells was evaluated by MTT assay.

### Mining Data and Microarray Data Processing

Publicly available data from 10 different tumor data sets, profiled on Affymetrix platforms deposited in the Gene Expression Omnibus (GEO); RNA sequencing data available at The Cancer Genome Atlas (GDC TCGA Breast Cancer) from the Xena browser (22); and normalized data from METABRIC study (23), recovered from cBioportal (24), were downloaded, and a total number of 1,320 TNBC tumors were evaluated. ER, PgR, and HER2 immunohistochemistry status was used to define the triple-negative phenotype. Supplementary Table 1 contains relevant characteristics of the data sets analyzed. All data retrieved from GEO were downloaded as raw data (cel file) and processed as follows: Microarray data from U133A, U133 Plus 2.0 and human transcriptome arrays were independently normalized and background corrected using RMA and quantile algorithms implemented in oligo package from Bioconductor (25). Genes from each platform were annotated with biomaRt (26), and only common cross-platform genes (including data from TCGA and METABRIC cohorts) were selected for further analysis. Duplicated probes were collapsed by selecting the probe with the highest interquartile range across the evaluated samples. Normalized data were scaled by median-absolute-deviation (MAD) for each sample. TCGA data were downloaded as raw counts and processed with limma-voom in limma R package (27).

### Tumor Purity

Tumor purity was inferred according to the ESTIMATE algorithm (28). We applied the formula to the normalized MAD values and for limma-voom transformed data from TCGA tumors. Because some samples presented low tumor composition, we restricted our analysis to samples with an inferred tumor purity value over 60%.

### Adjusted CTLA-4 Expression

To reduce bias due to remainder immune contribution to CTLA-4 expression levels, we adjusted it through a lineal regression model:

Adjusted CTL4 expression = lm(CTLA4~Est+CD4^*^CD8).

### GSEA and ssGSEA

Non-preranked gene set enrichment analysis (GSEA) (29) was carried out via local software and Molecular Signatures collection (MSigDB: hallmarks, KEGG, Biocarta, Reactome, and Gene Ontology Biological Process Sets). GSEA was computed on the common cross-platform genes of the studied cohorts adjusted through median centering. Significantly enriched gene sets were reported based on NES and FDR value (<0.25). Single-sample GSEA (ssGSEA) (30) was performed with previously published gene signatures or gene sets from MSigDB, using GSVA Bioconductor package (31). For activated-like phenotype, we analyzed the MSigDB sets: for ERK1/2, (1) GO positive regulation of ERK1 and ERK2 cascade, (2) Reactome ERK MAPK targets, and (3) GO ERK1 and ERK2 cascade; for AKT pathway, (1) Hallmark PI3K/AKT/mTOR signaling, (2) Reactome AKT phosphorylated targets in the cytosol, and (3) Reactome AKT phosphorylated targets in the nucleus (Supplementary Table 2). The gene list of immune signatures was summarized by Charoentong et al. (32).

### Immune Infiltration Characterization

The CIBERSORT web tool (https://cibersort.stanford.edu) was used to infer the relative proportions of immune cells using the leukocyte gene signature matrix (LM22) to deconvolve our MAD normalized datasets with 1,000 permutations (33). Relative proportion values were plotted in a heatmap. Additionally, xCell tool using default parameters (http://xcell.ucsf.edu/) was used to confirm immune infiltration composition and contribution of stem cells compartment. MAD-normalized gene expression data was analyzed. Normalized cell enrichment scores (ratio between the enrichment score of each sample and the maximum value among all samples) were evaluated.

### Lehmann Subtypes

TNBC subtypes described by Lehmann et al. (34) were inferred via the web-based tool “TNBC type” (http://cbc.mc.vanderbilt.edu/tnbc/) based on MAD-normalized gene expression.

### Characterization by Gene Signatures

Proliferation signature was recovered from the literature (proliferation Gene Cluster UNC337 intrinsic clustering) (35), and mean expressions of the multiple genes were computed as the score value. Cytolytic activity score has been defined as the mean of GZMA and PRF1 normalized gene expression (36). Tumor Inflammation Signature (TIS) score was defined by the average mean of normalized expression of the identified genes (37). Immune-related signatures were computed by the average gene expression value of each identified signature, as described previously (38, 39). To define responder patients based on anti-CLTA-4 immune scores, a support vector machine (SVM) approach was conducted on R. To train the model, data from TCGA TNBC tumors reported in Supplementary Table 3 (TNBC, *N* = 148) (39) was used. After the model construction, benchmark datasets were analyzed in R.

### Statistical Analysis

Clinical data were analyzed using the SPSS™ Statistics Software (19.0 IBM Co, NY, USA). Differences in smoking history and treatment features between CTLA-4-expressing and CTLA-4-negative groups were analyzed by the Fisher's exact test. Chi-square test was applied to compare the expression of CTLA-4 among groups with different tumor stage at diagnosis. Results of *in vitro* experiments were presented as the mean ± standard error (SEM). *T*-test was used to compare the *in vitro* treatment groups and cell lines. Confidence intervals (CI; 95%) and *p*-values were calculated. Two-tailed *p*-values < 0.05 were considered statistically significant. Data were plotted using ggplot in R environment and GraphPad Prism software (GraphPad software). The statistical differences between the different conditions from benchmark TNBC data were assessed by Wilcoxon test taking HH condition as reference (^*^p-value ≤ 0.05; ^**^p-value ≤ 0.01; ^***^p-value ≤ 0.001; ^****^p-value ≤ 0.0001). Fisher's exact test and Benjamin-Hochberg adjusted *p*-values were computed to examine the distributions of Lehmann subtypes and responder vs. non-responder groups.

## Results

### Tumor Cells of TNBC Patients Express CTLA-4 in Different Cell Compartments

**Figure 1:**
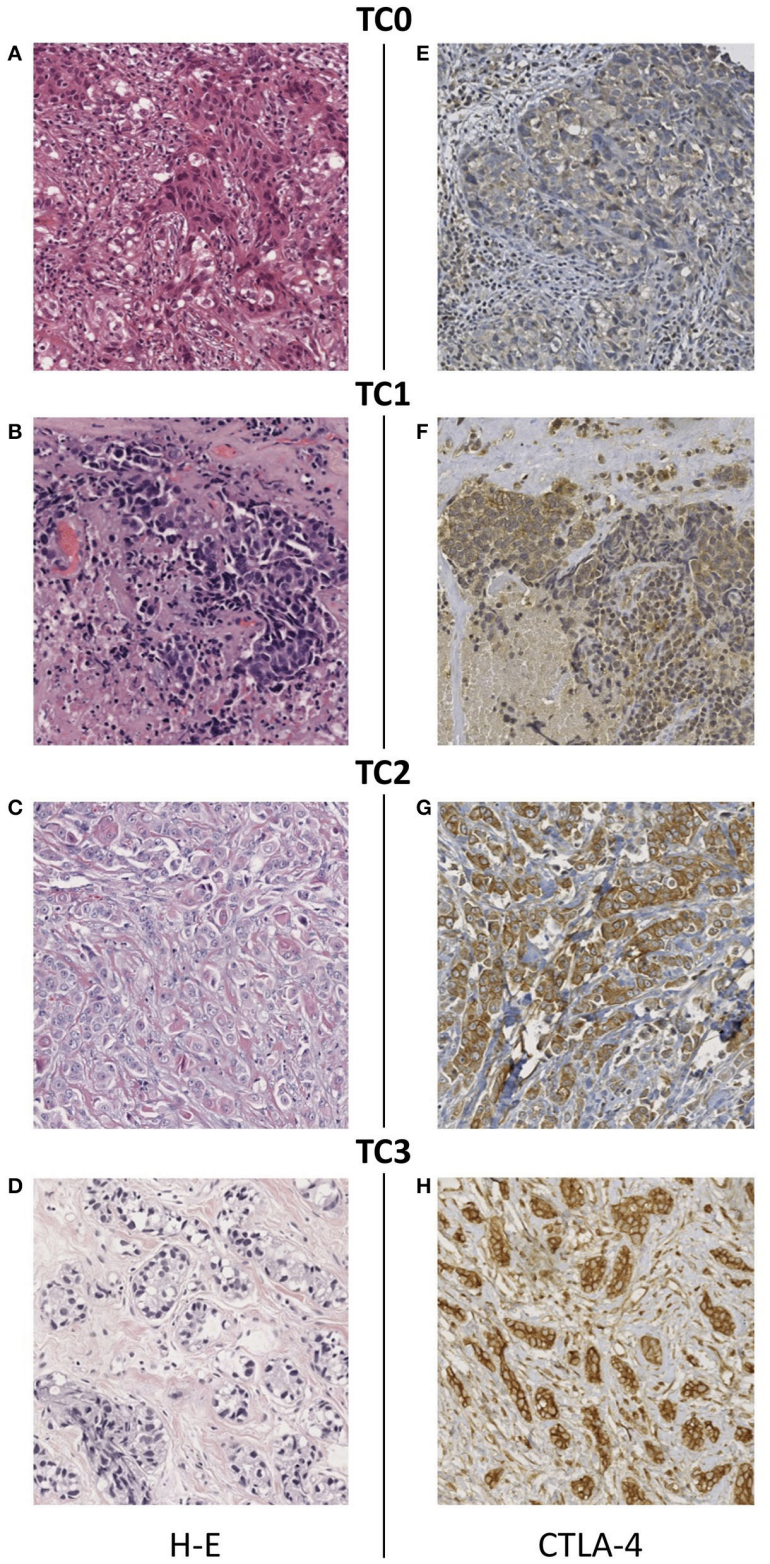
Immunohistochemical staining of CTLA-4 in TNBC tissues. **(A–D)** Representative images of hematoxylin-eosin staining and CTLA-4 membrane staining showing different intensity scores: **(E)** TC0 (<1%), **(F)** TC1 (>1 and <5%), **(G)** TC2 (>5 and <50%), **(H)** TC3 (>50%). All images are 20 × magnification.

A total of 50 TNBC cases were included. At diagnosis, 34% of patients had a tumor in early stage, 48% were locally advanced cases, and 14% presented metastatic disease. Complete clinicopathological data of the study population is summarized in Table 1. Expression of CTLA-4 was evaluated in tumor cells and infiltrating lymphocytes of TNBC paraffin-embedded biopsies, using an antibody that was previously validated in a breast cancer study (40). The quality of all tumor tissues was examined in H-E-stained slides (Figures 1A–D). All tumors showed cytoplasmic CTLA-4 staining, whereas different staining intensities were observed at the cell membrane. Overall, 15 tissue samples were negative (<1%) for CTLA-4 membrane staining, and they were classified as TC0 (Figure 1E). Surface CTLA-4 immunostaining was detected in 70% (35/50) of TNBC biopsies; 14% (7/50) of them showed a low/weak expression, scored as TC1 (>1 and <5%) (Figure 1F); 42% (21/50) showed a medium expression, scored as TC2 (>5 and <50%) (Figure 1G); and 14% (7/50) showed a high/strong membrane expression, scored as TC3 (>50%) (Figure 1H). Immunohistochemistry analysis also showed positive CTLA-4 immunostaining in lymphocytes of samples presenting tumor-infiltrating lymphocytes (TILs) (Table 1).

Categorization of samples by tumor stage showed that the proportion of samples with expression of CTLA-4 at the cell-surface did not differ among patients diagnosed with early, locally advanced, and metastatic tumors (Fisher's Exact Test, *p* = 0.535) (Table 2). Expression of CTLA-4 was not significantly associated with history of smoking (*p* = 0.546) or treatment (neoadyuvant vs. non-neoadyuvant chemotherapy, *p* = 0.071).

**Table 2:** Association of CTLA-4 expression with tumor stage, smoking history, and chemotherapy.

	**TC0** ***N* (%)**	**TC1** ***N* (%)**	**TC2** ***N* (%)**	**TC3** ***N* (%)**	p^*^
**Stage**					
Early	3 (17.65)	2 (11.76)	9 (52.94)	3 (17.65)	0.535
Locally advanced	8 (33.33)	4 (16.67)	11 (45.83)	1 (4.17)	
Metastasis	2 (28.57)	1 (14.29)	1 (14.29)	3 (42.86)	
**Smoking**					
Yes	7 (35.00)	4 (20.00)	6 (30.00)	3 (15.00)	0.546
No	8 (26.67)	3 (10.00)	15 (50.00)	4 (13.33)	
**Chemotherapy**					
Neoadyuvant	11 (42.31)	3 (11.54)	10 (38.46)	2 (7.69)	0.071
Non-neoadyuvant	4 (17.39)	4 (17.39)	11 (47.83)	4 (17.39)	

* *Fisher's Exact Test comparing all CTLA-4-expressing (TC1, TC2, and TC3) vs. CTLA-4-negative (TC0) case*.

### CTLA-4 and Its Primary Ligands CD80/CD86 Are Expressed on TNBC Cell Lines and Tumors

**Figure 2:**
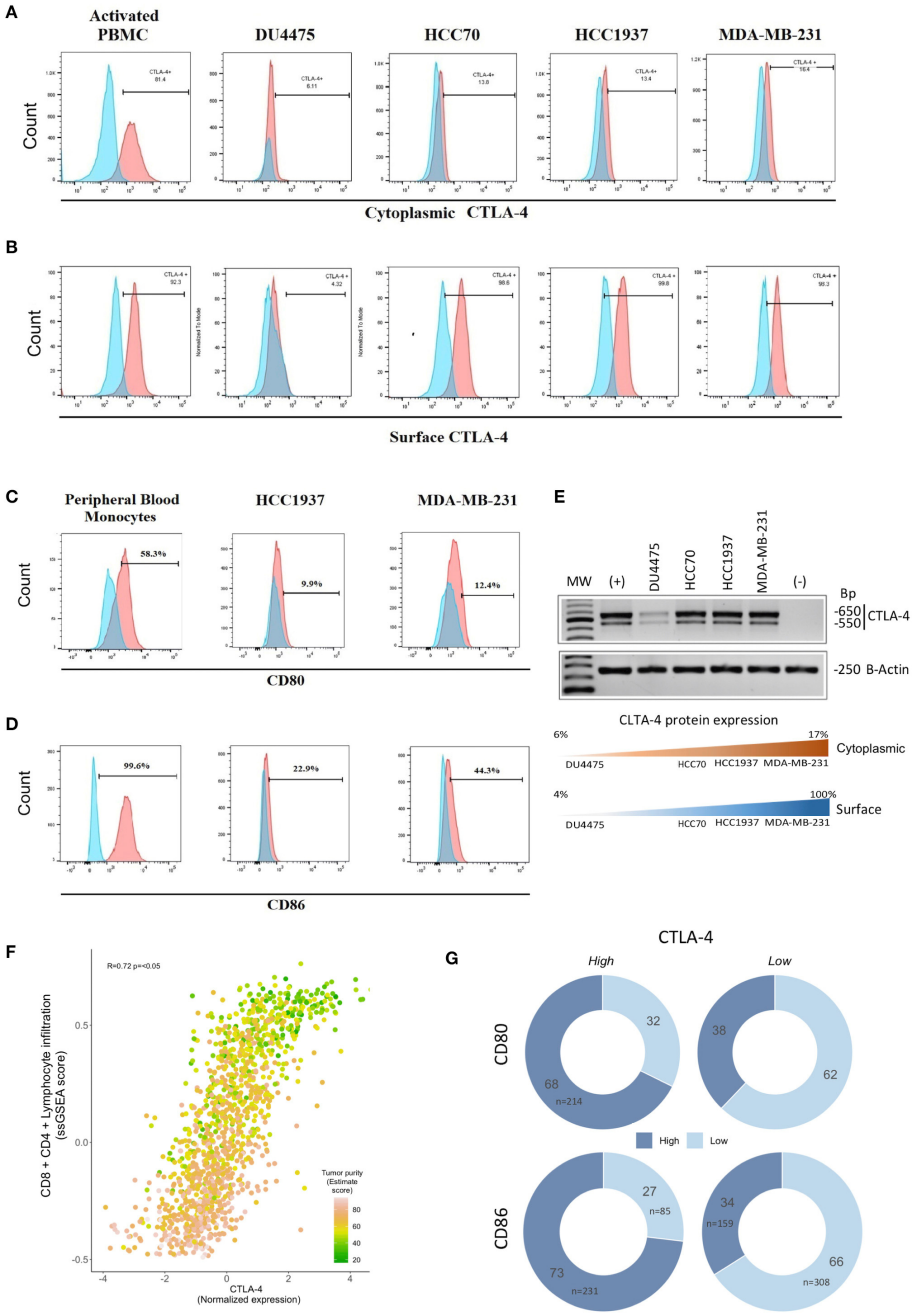
Expression of CTLA-4, CD80/CD86 ligands, and CTLA-4-associated pathways in TNBC cell lines. Expression of CTLA-4 protein was evaluated by flow cytometry using phytohemagglutinin-L-activated human lymphocytes (PBMC) as a positive control in **(A)** intracellular compartment and **(B)** cell-surface compartment. Expression of cell-surface **(C)** CD80 and **(D)** CD86 in HCC1937 and MDA-MB-231 cells by flow cytometry, including *in vitro*-cultivated human peripheral blood monocytes as a positive control. **(E)** The expression of the CTLA-4 gene was investigated by RT-PCR in the DU4457, HCC70, HCC1937, and MDA-MB-231 cell lines. Phytohemagglutinin-L-activated human lymphocyte RNA was included as a positive control (+), and RNA from *in vitro*-cultured human peripheral blood monocytes as a negative control (–). Lower panel: Representation of protein expression results **(F)** Scatterplot showing the correlation between CTLA-4-normalized expression and ssGSEA-enriched score of immune signatures (CD4/CD8, lymphocyte infiltration) among human triple-negative tumors from public databases (*N* = 1,320). CTLA-4 expression was highly concordant with lymphoid infiltration and tumor purity (Spearman correlation *R* = 0.72; *p* < 0.05). Colors of each point represent tumor purity values inferred by the ESTIMATE algorithm. **(G)** Donut charts showing the distribution of TN tumors over-expressing CTLA-4/CD80/CD86 (mRNA level). Color shades indicate high or low CD80 or CD86.

Our previous results demonstrated the expression of CTLA-4 on the membrane of TNBC tissue. Thus, we next analyzed the expression of CTLA-4 in TNBC-derived cell lines (DU4475, HCC70, HCC1937, and MDA-MB-231). The intracellular expression of CTLA-4 was confirmed by flow cytometry and was observed in all evaluated cell lines. The proportion of intracellular CTLA-4 positive cells ranged from 6.11 to 16.4%. The MDA-MB-231 cell line presented the highest proportion of intracellular CTLA-4 (16.4%) (Figure 2A). Since only CTLA-4 expressed on the cell surface is functional (41), we next analyzed membrane expression of CTLA-4. Membrane CTLA-4 was observed in all cell lines. The proportion of cells expressing membrane CTLA-4 was higher than 90% in HCC70, HCC1937, and MDA-MB-231 cell lines, while only 4.3% of DU4475 cells expressed CTLA-4 in this compartment (Figure 2B).

CTLA-4 activation occurs by interaction with its primary ligands CD80/CD86 on antigen-presenting cells (42–44). Thus, we next investigated the expression of CD80 and CD86 on the surface of HCC1937 and MDA-MB-231 cells. Flow cytometry analysis showed that CD80 was weakly expressed in both cell lines (<13% of positive cells), compared to CD86, which was detected in 22.9% of HCC1937, and 44.3% of MDA-MB-231 (Figures 2C,D), suggesting a potential interaction of CD80/CD86-expressing cells with CTLA-4-expressing cells that might activate the receptor.

To extend our observations, we collected a comprehensive benchmark dataset evaluating gene expression profiles of 1,320 TNBC tumors. To assess a surrogate measurement of CTLA-4 protein expression based on mRNA levels, we first analyzed the presence of CTLA-4 mRNA in four TNBC-derived cell lines by RT-PCR. CTLA-4 mRNA was detected in all TNBC cells (Figure 2E). The amplified fragments observed were of the same size as those detected in activated human leukocytes, and a good relationship between CTLA-4 protein and mRNA level was observed. This suggests that it is suitable to use CTLA-4 gene expression as a surrogate value of protein status (Figure 2E).

Since a major source of CTLA-4 in TNBC tumors is the infiltrated lymphocyte immunome, we computed CD8/CD4 lymphocyte infiltration score, determined through ssGSEA of publicly available signatures (32) and compared it to CTLA-4 gene expression levels. Indeed, we observed a highly significant correlation of CTLA-4 gene expression and the CD8/CD4 enrichment score (*N* = 1,320, *R* = 72%, *p* < 0.05) (Figure 2F). To guarantee robust power to detect tumor CTLA-4 expression by assuring the highest tumor cell content, we selected tumors with at least 60% tumor purity, defined by the ESTIMATE algorithm (*N* = 783, Supplementary Table 1). Moreover, to minimize the bias due to the contribution of infiltrating lymphocytes, the reported CTLA-4 level was adjusted by the ssGSEA CD8/CD4 infiltration score and purity estimated value, ensuring a minimal impact of the immune compartment on the evaluated CTLA-4 expression. Notably, when looking at the proportions of TNBC over-expressing CTLA-4, 73% were found up-modulated in CD86 and 68% in CD80, whereas tumors with low levels of CTLA-4 generally presented a lower frequency of CD86 (34%) and CD80 (38%) up-modulation (Figure 2G). Taken together these observations indicate that the coordinated expression of the CTLA-4/CD80/CD86 axis is a widespread feature of TNBC.

### Transcriptional Landscape of CTLA-4-Expressing Tumors Is Related to Relevant Oncogenic Pathways

It is known that activation of CTLA-4 on T-cells inhibits the PI3K/AKT pathway (45–47). Thus, to investigate the effect of CTLA-4 on the activation of the PI3K/AKT cascade, HCC1937 and MDA-MB-231 cells were incubated with either recombinant CD80 or Ipilimumab. Activation of AKT by phosphorylation of the Thr308 or Ser473 residues was evaluated at different time points by Western blot. Our analysis showed constitutive phosphorylation of the Ser473 residue in HCC1937 cells, which was significantly increased after incubation with Ipilimumab, but experienced no alteration after incubation with CD80.

**Figure 3:**
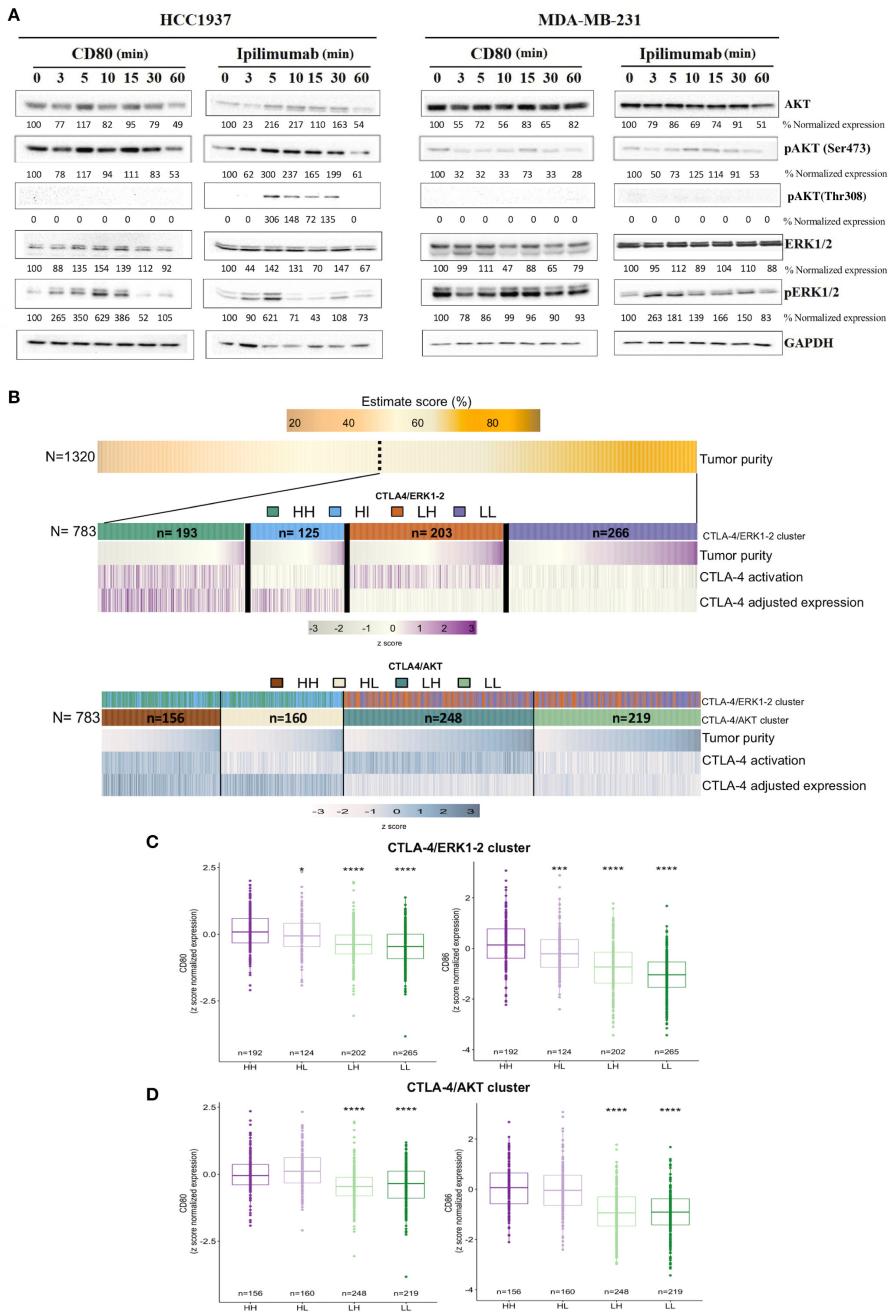
Transcriptional landscape of activated-like CTLA-4 in human tumors from publicly available data. **(A)** Evaluation of AKT and ERK1/2 activation signaling pathways after incubation with either CD80 (0.15 μg/ml) or Ipilimumab (10 μg/ml) at the indicated time points. Activation of AKT was evaluated by Western blot using antibodies against phosphorylated Ser473 (pAKT-Ser473) and Thr308 (pAKT-Thr308) residues. Activation of ERK1/2 was analyzed by using an antibody that recognizes phosphorylated Thr185 + Thr202 + Tyr204 + Tyr187 residues (pERK1/2). Expression of GAPDH was included as loading control. Densitometric analysis indicated as percentage below each blot, taking time 0 as reference for each evaluated protein. **(B)** Heatmap of decoding tumor subgroups based on CTLA-4 expression and activation. First panel shows the inferred tumor purity value (%) among the complete TNBC cohort (*N* = 1,320). The following panels display selected tumors based on tumor purity above 60% (*N* = 783). Middle panel shows tumor subtype features of CTLA-4 ERK1-2 tumors, while lower panel shows features of CTLA-4 AKT tumors. Samples represented in the plot were sorted by tumor purity value in each cluster. CTLA-4 expression value was adjusted by CD8/CD4/lymphocyte infiltration and ESTIMATE value. Boxplots of CD80 and CD86 normalized expression values in **(C)** CTLA-4/ERK cluster and **(D)** CTLA-4/AKT cluster (HH: CTLA-4^H/H−ERK1/2^ and CTLA-4^H/H−AKT^; HL: CTLA-4^H/L−ERK1/2^ and CTLA-4^H/L−AKT^; LH: CTLA-4^L/H−ERK1/2^ and CTLA-4^L/H−AKT^; LL: CTLA-4^L/L−ERK1/2^ and CTLA-4^L/L−AKT^). (*p-value ≤ 0.05; ***p-value ≤ 0.001; ****p-value ≤ 0.0001).

Conversely, incubation with CD80 induced a significant reduction of Ser473 phosphorylation in MDA-MB-231 cells, whereas Ipilimumab had no effect on the phosphorylation of this residue (Figure 3A). In sharp contrast, we observed that phosphorylation of the Thr308 was absent in both cell lines. Incubation with Ipilimumab resulted in the phosphorylation of Thr308 in HCC1937 cells but not in MDA-MB-231. No changes were observed after CD80 incubation. These observations suggest that blockade of CTLA-4 induces full activation of AKT through the phosphorylation of Ser473 and Thr308 in HCC1937 cells. It has also been described that CTLA-4 is able to activate the MAP kinase ERK1/2 pathway (48). Thus, we analyzed the effect of activating or blocking CTLA-4 on the phosphorylation of ERK1/2. As shown in Figure 3A, ERK1/2 is constitutively activated in MDA-MB-231 cells and phosphorylation was not affected by any of the treatments. In contrast, incubation of HCC1937 cells with CD80 induced the phosphorylation of ERK1/2 after 3 min of treatment. ERK1/2 remained phosphorylated for up to 15 min. When the cells were incubated with Ipilimumab, a modest phosphorylation of ERK1/2 was observed. However, it was only detected in early time points, at 3 and 5 min post-incubation. We demonstrated that CTLA-4 expressed on breast cancer cells, mainly in the membrane of HCC1937 cell line, is functional and induces the activation of the MAP kinase pathway, while AKT signaling is abrogated.

To study the role of CTLA-4 on TNBC tumors, we explored public data with a tumor purity >60%. Tumors showing a CTLA-4 expression values over the median were assumed to be CTLA-4^High^ (CTLA-4^H^). Then, to infer CTLA-4 activation by cell-membrane expression, the results of *in vitro* experiments were considered, and tumors were subjected to ssGSEA analysis using the enrichment scores of ERK1/2 and its target gene set, as well as PI3K/AKT/mTOR and AKT target gene sets (nucleus and cytoplasm). Four major groups were defined for each oncogenic pathway. The resulting clusters were described based on the ERK1/2 pathway as follows: tumors that are CTLA-4^H/H−ERK1/2^ (*N* = 193), representing the biological relevant group for our study where tumor cells over-express CTLA-4 and are activated-like tumor as shown by ERK1/2 enrichment pathway; CTLA-4^H/L−ERK1/2^ (*N* = 125), which probably do not express CTLA-4 at the membrane level; and two low CTLA-4 expression groups, CTLA-4^L/H−ERK1/2^ (*N* = 203) and CTLA-4^L/L−ERK1/2^ (*N* = 226) (Figure 3B). Based on the AKT pathway, tumors were categorized as: CTLA-4^H/H−AKT^ (*N* = 156), which do not express membrane CTLA-4, and CTLA-4^H/L−AKT^, representing our study phenotype where CTLA-4 is up-regulated in tumor cells and is activated-like, as defined by the down-modulation of the AKT pathway. Finally, CTLA-4^L/H−AKT^ and CTLA-4^L/L−AKT^ were also detected (Figure 3B).

To explore whether expression of the CTLA-4/CD80/CD86 axis was associated with an activated-like phenotype, gene expression of CD80/CD86 was evaluated on TNBC. CTLA-4^H/H−ERK1/2^ tumors presented upregulation of CD80 (Figure 3C), but a more significant over-expression of CD86 compared to CTLA-4^H/L−ERK1/2^ samples (Figure 3D). This observation recapitulates the results of our *in vitro* assays and suggests the presence of active CTLA-4/CD80/CD86 paracrine signaling.

**Figure 4:**
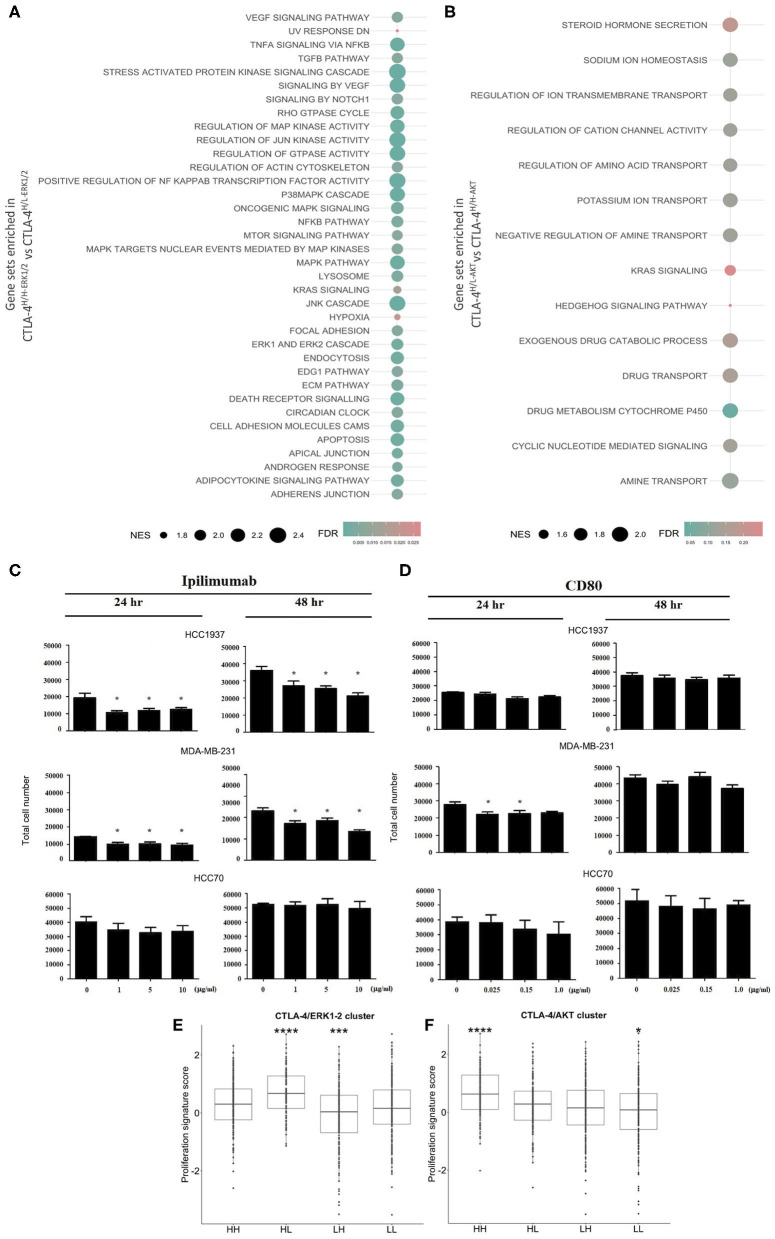
Biological effects of CTLA-4 activation in human tumors and *in vitro* cell line models. Results from GSEA as bubble plot comparing **(A)** CTLA-4^H/H−ERK1/2^ vs. CTLA-4^H/L−ERK1/2^ and **(B)** CTLA-4^H/L−ERK1/2^ vs. CTLA-4^H/H−ERK1/2^, where the size of the circles gives the normalized enrichment score (NES) and the color Indicates FDR. *In vitro* assays to evaluate the effect of the activation and blockade of CTLA-4 on cell proliferation. **(C)** The effect of blocking CTLA-4 on the proliferation of HCC1937 and MDA-MB-231 cells was analyzed by incubating cells with the monoclonal antibody Ipilimumab (10.0 μg/ml), cell number was evaluated at 24 and 48 h post-incubation. **(D)** The effect of activation of CTLA-4 on the proliferation of HCC1937 and MDA-MB-231 cells was analyzed by incubating cells with recombinant human CD80 (0.15 μg/ml), cell number was evaluated at 24 and 48 h post-incubation. Boxplots for the expression of proliferation gene signatures in **(E)** CTLA-4/ERK1-2 and **(F)** CTLA-4/AKT cluster. (*p-value ≤ 0.05; ***p-value ≤ 0.001; ****p-value ≤ 0.0001.

To get insight into the transcriptional landscape of the categorized CTLA-4 tumors, a GSEA analysis was performed based on common cross-platform genes evaluated by the public expression profiles of TNBC tumors. Several potentially actionable molecular alterations were enriched in CTLA-4-over-expressing activated-like tumors. We initially focused on the comparison between ERK-activated tumors (CTLA-4^H/H−ERK1/2^ vs. CTLA-4^H/L−ERK1/2^). As expected, GSEA denoted the enrichment of ERK1/2 down-stream signaling pathways such as TGF-β, multiple signal transduction processes, such as tumor necrosis factor α (TNFα), extracellular-signal-regulated kinases (ERKs), cJun kinases (JNKs), and GTPase activity in CTLA-4^H/H−ERK1/2^ tumors. GSEA also pointed out pathways activated by TNFα including apoptosis, NF-κB, and MAP kinases (Figure 4A and Supplementary Table 3). Other enriched processes in this tumor phenotype included KRAS, a critical regulator of oncogenic properties, cell–cell adhesion, androgen feedback, and stress responses to hypoxia and UV exposure (Figure 4A and Supplementary Table 3). Hypoxic conditions result in the transcription of various cytokines, including VEGF, which is a pro-angiogenic factor and was enriched in CTLA-4^H/H−ERK1/2^ tumors. Some pathways overlap with CTLA-4-activated-like tumors with an abrogated AKT program (CTLA-4^H/L−AKT^ vs. CTLA-4^H/H−AKT^), such as KRAS signaling and stem-like related pathways including Notch and Hedgehog. GSEA also revealed unique significant enrichment of gene sets related to drug metabolism and regulation of ion and amino acid transport (Figure 4B).

As a negative regulator of T cell activity, alterations of AKT and ERK1/2 cascades induced by CTLA-4 have been associated with the inhibition of cell proliferation (49, 50). Therefore, we investigated whether incubation of CTLA-4-expressing cells with various concentrations of recombinant CD80 or with Ipilimumab might regulate proliferation rates at different time points. As shown in Figure 4C, blockade of CTLA-4 by incubation with Ipilimumab induced a dose-dependent reduction of HCC1937 and MDA-MB-231 cell numbers 48 h post-treatment (*p* < 0.01), but not in HCC70 cells. CD80 incubation only modified the number of cells at early time points (24 h) in MDA-MB-231, but not at 48 h (Figure 4D). Incubation with CD80 had no effect on the proliferation of HCC70 and HCC1937 cells. Further inspection of proliferation among TNBCs revealed that CTLA-4-activated-like tumors present lower expression of proliferation gene clusters in comparison with tumors that only over-expressed the immune checkpoint (Figures 4E,F). Although the GSEA analysis indicated the significant enrichment of potential cell adhesion regulators, which may impact cell motility/invasion, and could be associated with mesenchymal features, we were not able to demonstrate an effect of CTLA-4 modulation on cell invasion (Supplementary Figure 1A) or on the expression of epithelial-to-mesenchymal biomarkers, *in vitro* (Supplementary Figure 1B).

Together, these results revealed a unique transcriptomic landscape of CTLA-4-activated-like tumors that might impact relevant cancer programs locally, in the tumor cell, and in the tumor microenvironment.

### CTLA-4-Activated Tumors Are Enriched in Immunogenic Pathways and CTLA-4 Blockade Increases IL-2 Secretion

**Figure 5:**
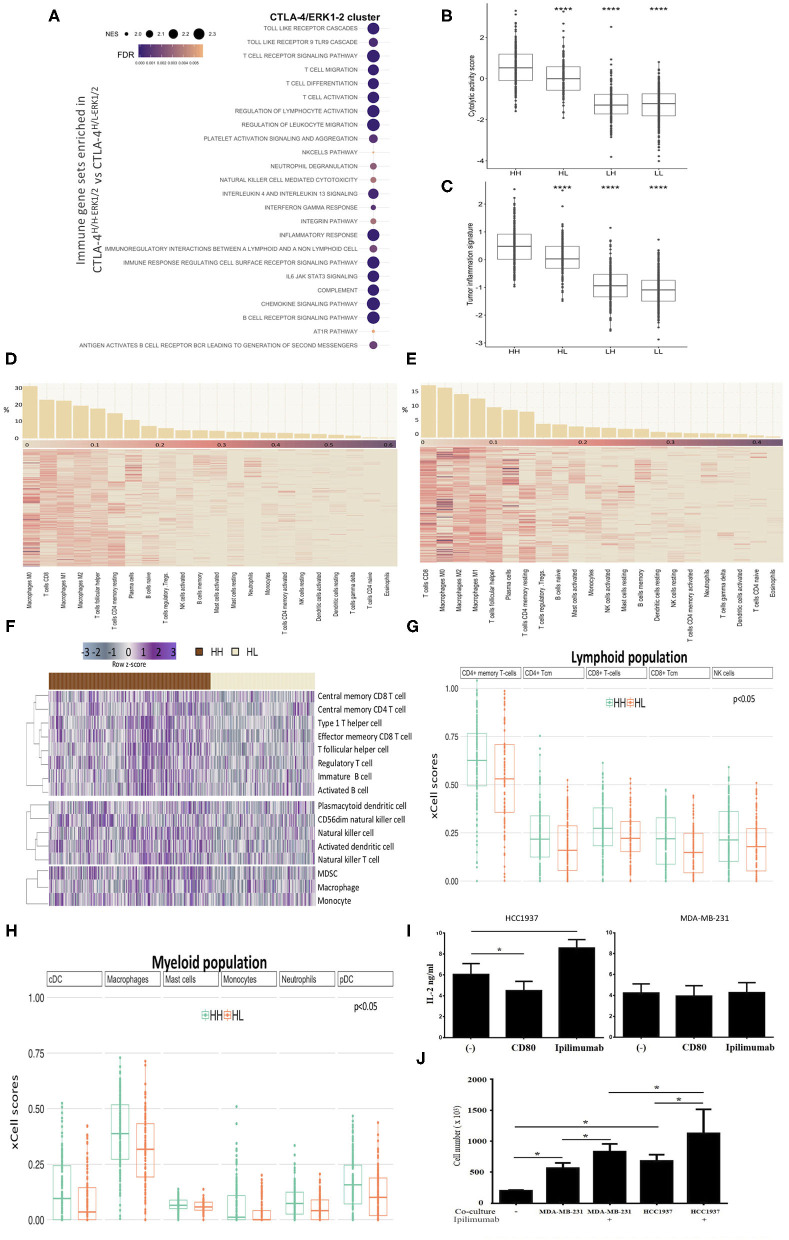
Immunophenotypes of TNBC with activated CTLA-4. **(A)** Bubble plot showing the most significant enriched immunological gene sets from GSEA in CTLA-4^*H*/*H*−*ERK*1/2^ compared to CTLA-4^*H*/*L*−*ERK*1/2^ tumors. Bubble size represents normalized enrichment score (NES) and color FDR value (<0.05). Box plots of **(B)** cytolytic activity (CYT) and **(C)** Tumor Inflammation Signature (TIS) according to gene signatures among TNBC (HH: CTLA-4H/H-ERK1/2; HL: CTLA-4H/L-ERK1/2; LH: CTLA-4L/H-ERK1/2; LL: CTLA-4L/L-ERK1/2). Heatmap of relative proportions of immune subpopulations computed with CIBERSORT in **(D)** CTLA-4H/H-ERK1/2 and **(E)** CTLA-4H/L-ERK1/2 tumors. Samples are sorted according to the relative proportion of each immune cell (Barplots in upper panel). **(F)** Heatmap of significant enriched ssGSEA scores (FDR < 0.05) of immune cell subpopulations according to immune signatures described by Charoentong in CTLA-4H/H-ERK1/2 TNBC compared to CTLA-4H/L-ERK1/2. Upper: adaptive immunity, middle: adaptive-innate immunity, and lower: innate immunity. Boxplots showing the xCell scores of **(G)** Lymphoid population scores and (H) Myeloid population scores differently distributed (FDR < 0.05) within HH (CTLA-4H/H-ERK1/2) and HL (CTLA-4H/L-ERK1/2) tumors. **(I)** Secretion of IL-2 by HCC1937 and MDA-MB-231 was evaluated by quantitative ELISA after incubation with CD80 (0.15 μg/ml) or Ipilimumab (10 μg/ml). **(J)** Effect of CTLA-4 blockade on lymphocytes proliferation (phytohemagglutinin-stimulated peripheral blood leukocytes) in co-culture with HCC1937 and MDA-MB-231 cells in the presence and absence of Ipilimumab. (*p-value ≤ 0.05; ****p-value ≤ 0.0001).

Rationalizing that tumor expression of CTLA-4 not only regulates cellular functions, but may also modulate the immune microenvironment, we inspected the immunogenicity characteristics of cell lines and tumors. First, GSEA revealed an enrichment of immune-related pathways only in the CLTA-4-activated-like phenotype, inferred by ERK status (Figure 5A and Supplementary Table 3). This analysis showed that CTLA-4-activated tumors are highly enriched in immune cells (T cells, NK cells, neutrophils) and immune pathways (immune activation, interleukin and immune communication signaling) with relevant regulatory functions.

Considering that physiological anti-tumor immunity requires a cytolytic immune response, cytolytic activity scores (CYT) (36) were computed in TNBC datasets. CTLA-4^H/H−ERK1/2^ tumors exhibited significantly higher CYT scores compared to CTLA-4^H/L−ERK1/2^ (*p* = 0.0001) (Figure 5B). Furthermore, a pre-existing adaptive immune response within tumors was inferred via the Tumor Inflammation Signature (TIS) algorithm (37); a similar enriched profile was found for CTLA-4^H/H−ERK1/2^ tumors, which presented the highest TIS score (*p* = 0.0001) (Figure 5C).

To define the crosstalk between the activated-CTLA-4 axis in tumor cells and the intra-tumor immune infiltrate, we estimated the immune cellular composition of the analyzed TNBC tumors based on their transcriptome profiles, through dedicated algorithms. First, we estimated the relative immune cellular composition using the CIBERSORT deconvolution method (33). We observed that the frequency of the immunological sub-populations appears to be distinct for each tumor subtype (Figures 5D,E and Supplementary Table 4). We then validated the predicted immune composition based on benchmark signatures (32) by ssGSEA analysis. We identified 16 kinds of immune cell subtypes that had different enrichment scores between CTLA-4^H/H−ERK1/2^ and CTLA-4^H/L−ERK1/2^. Five adaptive immune cells were significantly associated with the CTLA-4^H/H−ERK1/2^ phenotype (Figure 5F), and these tumors also showed a significant up-regulation of three classes of NK cells and two dendritic cells. A high degree of consistency with immune scores estimated by xCell (51) in CTLA-4^H/H−ERK1/2^ tumors vs. CTLA-4^H/L−ERK1/2^ was observed (Figures 5G,H and Supplementary Table 4). xCell analysis also pointed out the loss of stem cell compartment, particularly the mesenchymal stem cell (MSC) population and common lymphoid progenitors (CLP) on CTLA-4-activated-like tissues (Supplementary Figure 2A).

In agreement with the above described results, ERK-activated-like tumors over-expressing CTLA-4 (CTLA-4^H/H−ERK1/2^) were enriched in immune (IM) and basal-like 1 (BL1) subtypes, as described by the TNBC molecular classification defined by Lehmann (34), in comparison to down-modulating CTLA-4 tumors [Fisher's exact, Benjamin–Hochberg (BH) *p* < 0.05] (Supplementary Figure 2B). Additionally, no differential enrichment was observed in IM or BL1 subtype distribution in CTLA-4^H/L−AKT^ and CTLA-4^H/H−AKT^ tissues, corroborating the results computed through the GSEA (Supplementary Figure 2C).

A major effect of CTLA-4 activation on lymphocytes is the inhibition of interleukin IL-2 production and secretion (49, 50). Thus, to address the question of whether CTLA-4 modifies IL-2 secretion by TNBC cells, the concentration of soluble IL-2 in culture medium of untreated and cells treated with either recombinant CD80 or Ipilimumab was evaluated by ELISA. Incubation with CD80 significantly reduced IL-2 secretion by HCC1937 cells, but had no effect on MDA-MB-231 cells (Figure 5I). In contrast, incubation with Ipilimumab markedly enhanced IL-2 secretion on HCC1937 cells (Figure 5I), while MDA-MB-231 cells were unaffected (Figure 5I). These observations suggest the potential effects of CTLA-4 activation on IL-2 secretion in some TNBC cells.

Since IL-2 is a critical stimulator of T cell proliferation and differentiation, we next investigated whether proliferation of lymphocytes can be modulated by TNBC cell lines secreting IL-2. Co-culture of lymphocytes with tumor cells induced a significant increment on the number of viable lymphocytes (*p* < 0.05), which may be explained by the natural production of IL-2 by both cell lines (Figure 5J). Further, blocking CTLA-4 with Ipilimumab increased the number of viable lymphocytes compared to non-treated cells (*p* < 0.05). The most significant effect was observed in lymphocytes co-cultured with HCC1937, which is consistent with the observation that Ipilimumab boosts IL-2 secretion in this cell line (Figure 5J). Together, these observations suggest that CTLA-4 blockade in tumor cells may contribute to generating a favorable microenvironment for immune responses.

Although IL-2 plays an important role in the expansion of cytotoxic T cells, recent studies have demonstrated that IL-2 is also crucial for the establishment and maintenance of T regulatory cells (Tregs) (52, 53). Thus, we explored the presence of Tregs in CTLA-4-expressing TNBC. Interestingly, application of deconvolution methods showed that tumors of the CTLA-4-activated subtype displayed a greater range of CD8/CD4 signatures, but we did not observe a differential enrichment of Tregs between the CTLA-4^H/H−ERK1/2^ and CTLA-4^H/L−ERK1/2^ tissues (Supplementary Figure 2D). Accordingly, the expression of FOXP3, a regulatory element necessary for Treg development (54), did not vary across CTLA-4 over-expressing tumors (Supplementary Figure 2E) and a similar correlation was detected between CD8/CD4 enrichment score and FOXP3 in CTLA-4^H/H−ERK1/2^ and CTLA-4^H/L−ERK1/2^ tumors (Supplementary Figure 2F).

Moreover, our results showed the presence of NK cells in CTLA-4^H/H−ERK1/2^ tumors. NK cell proliferation is regulated by IL-15 (55), but studies of IL-15 expression in breast cancer are scarce (56). Thus, we analyzed the level of IL-15 mRNA in tumors. The level of IL-15 mRNA in CTLA-4^H/H−ERK1/2^ tumors was significantly higher than the observed in CTLA-^4H/L−ERK1/2^ tumors (*p* < 0.05) (Supplementary Figure 3A). Likewise, immunogenomic analysis demonstrated a significant correlation between IL-15 mRNA expression and the NK fraction (*R* = 0.34, *p* < 0.05) (Supplementary Figure 3B). IL-15 is mainly produced by macrophages after stimulation with Colony Stimulating Factor-1 (CSF-1). Accordingly, we observed that CSF-1 is highly expressed in CTLA-4^H/H−ERK1/2^ tumors (Supplementary Figure 3C).

Overall, characterization of the tumor infiltrated microenvironment supports the idea that there are different patterns of tumor immune response associated with CTLA-4 expression levels and activated-like status in tumor-cells.

### CTLA-4 Activated Tumors Present Highly Predicted Response Scores to Immunotherapy Based on Immune Signature

**Figure 6:**
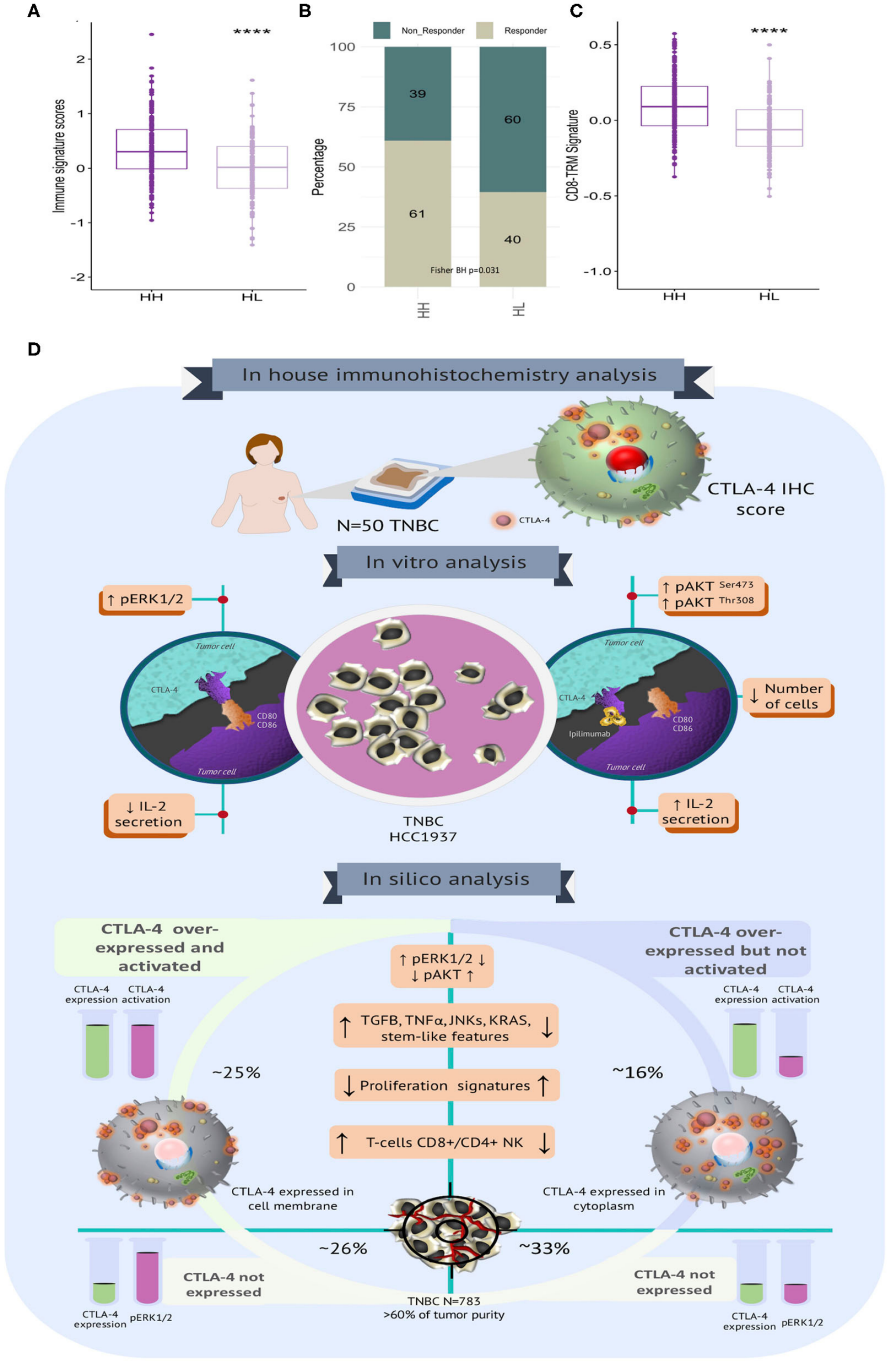
Immune signatures associated with immunotherapy response and clinical behavior in TNBC with diverse CTLA-4 portrait. **(A)** Boxplot of CTLA-4 immune signature scores according to tumor subtypes (HH: CTLA-4^H/H−ERK1/2^ and HL: CTLA-4^H/L−ERK1/2^). **(B)** Proportion of potential responders and non-responders to anti-CTLA-4 immunotherapy is shown according to tumor subtypes (Fisher BH *p*-value < 0.05). **(C)** Boxplot showing the distribution of CD8 TRM signature scores among TNBC stratified by CTLA-4 subtypes (HH: CTLA-4^H/H−ERK1/2^ and HL: CTLA-4^H/L−ERK1/2^) (****p-value ≤ 0.0001). **(D)** Graphical representation of the biological landscape of TNBC expressing CTLA-4. Immunohistochemistry (*N* = 50), preclinical *in vitro* evaluation (MDA-MB-231 and HCC1937) and *in silico* analysis of publicly available gene expression data on TNBC (*N* = 783).

Enhancing our understanding of the interaction between cancer cells and the immune system would lead to the improvement of immunotherapy for treating cancer (57–59). Thus, we aimed to assess the potential benefit of immunotherapy across TNBC benchmark data by interrogating their transcriptional profiles. We applied the Immune Score (IS) (39), which infers the response to anti-CTLA-4 immunotherapies in accordance with tumor CTLA-4 status. ISs were more elevated in CTLA-4^H/H−ERK1/2^ than in CTLA-4^H/L−ERK1/2^ tissues (Figure 6A). In accordance, the proportion of potential responders to CTLA-4 immunotherapy were highly enriched in CTLA-4^H/H−ERK1/2^ tumors (61% in CTLA-4^H/H−ERK1/2^ vs. 40% in CTLA-4^H/L−ERK1/2^, BH *p* = 0.031) (Figure 6B). To further dissect the biological role of the described immune phenotype, we assessed the tissue-resident memory T gene signature (TRM) (38), which described high enrichment of T cell populations in TNBCs and is able to distinguish patients with high CD8 expression who have a good or poor prognosis. TRM signature scores were higher in activated-CTLA-4 tissues (Figure 6C). Taken together, these results suggest that tumor cells over-expressing CTLA-4 may show distinct clinical and therapy responses as a result of the established biological signaling pathways.

## Discussion

CTLA-4 was initially described as a T cell-associated molecule. However, there is evidence showing the expression of CTLA-4 in various types of non-lymphoid cells (60). Here we describe the cytoplasmic and cell surface expression of CTLA-4 on TNBC samples and cell lines. Expression of CTLA-4 on breast cancer cells was first reported by Contardi et al. (15) in five ductal carcinoma patients with positive immunostaining at the membrane and cytoplasmic compartments, with similar intensity in all samples. Thereafter, the expression of CTLA-4 at protein and mRNA levels was demonstrated in breast cancer (60). More recently, CTLA-4 has been reported to be over-expressed in a high proportion of breast cancer samples (18). In contrast with former reports, we exclusively focused our study on the expression of CTLA-4 in TNBC. We observed that all analyzed TNBCs (*N* = 50) showed cytoplasmic expression of CTLA-4, as previously reported (61). However, differences in membrane expression were detected. Around 70% of tissues were positive for CTLA-4 at the membrane showing different levels of staining. Considering that only surface-expressed CTLA-4 is functional (41), these findings might have potential clinical implications. Unfortunately, the small number of patients included in the present work prevented us from correlating CTLA-4 surface expression and clinical features. Thus, evaluation of CTLA-4 expression in a larger TNBC population is necessary.

In agreement with earlier publications, we found that various TNBC-derived cell lines express CTLA-4 at the cell surface (14, 15). CTLA-4 is activated after binding to the CD80/CD86 ligands on the surface of antigen-presenting cells (62). Here, we detected the expression of CD80 and CD86 on the cell membrane of TNBC cell lines and human tumors, suggesting that this paracrine signal triggers CTLA-4 activation and its associated pathways.

Decoding the biological states of cell lines expressing CTLA-4 at the cell surface allowed us to detect a constitutive phosphorylation of the AKT-Ser473 residue in all cell lines, which was increased by Ipilimumab in HCC1937, but not in MDA-MB-231 cells. Surprisingly, we were not able to detect phosphorylation of the Thr308 residue in unstimulated cells. Nonetheless, incubation with Ipilimumab induced Thr308 phosphorylation in HCC1937 cells. In order to acquire full kinase activity, AKT must be phosphorylated in Ser473 at the C-terminal motif site and in Thr308 residue, which is located at the catalytic site (63, 64). Thus, it seems that CTLA-4 blockade may induce full activation of AKT signaling in particular cells, like HCC1937. Interestingly, AKT is a downstream target of PP2A, a crucial serine-threonine phosphatase, which associates to CTLA-4 and regulates its inhibitory activity (65), and reduced PP2A activity has been related with enhanced Thr308 phosphorylation, without altering Ser473, in acute myeloid leukemia (66). It was also reported that over-expression of CTLA-4 promotes the activation of the ERK1/2/MAPK pathway in different tumor types (67, 68) and stimulated T-cells (69). Additionally, coordinated anti-CTLA-4 and anti-PD-1 treatment suppressed the protein levels of phosphorylated ERK1/2 (70). Our results demonstrate that CD80 treatment induces the phosphorylation of ERK1/2 in HCC1937 cells at short post-treatment times.

We also systematically evaluated the transcriptional landscapes of publicly available gene expression profiles in accordance with CTLA-4 levels and their inferred activation statuses. Different contingency strategies to minimize bias due to the immune infiltrated compartment were applied to evaluate CTLA-4 mRNA portraits. We only studied samples with a predicted tumor purity higher than 60%, and CTLA-4 expression profiles were adjusted using the lymphocyte enrichment scores and tumor purity values. Our study decodes the heterogenous tumor clusters guided by CTLA-4 expression and activation and suggests the existence of diverse tumorigenic pathways in TNBC in association with CTLA-4 status. When the biological groups were stratified in accordance with their activated-CTLA-4 expression, we found that MAPK signaling, TGF-ß, VEGF, TNF-α, drug metabolism, ion and amino acid transport, and KRAS signaling were significantly associated with a CTLA-4 activated phenotype. Together, these observations indicate that CTLA-4 induces tumor driver pathways by distinct cellular mechanisms, primarily differing in ERK1/2 and AKT signaling processes (Figure 6D).

It is generally accepted that TNBC are tumors with a high rate of proliferation (71). Under this perspective, we analyzed the potential effect of CTLA-4 on proliferation rates. Molecular characterization of CLTA-4-activated-like tumors did not show differences in the expression of proliferation genes compared to tumors that only over-express CTLA-4. This could be partially explained by the reduced prevalence of Basal-like 1 subtype in the activated-like lesions, which are heavily enriched in annotations associated with proliferation (34). On the other hand, CTLA-4 blockade with Ipilimumab decreased total cell number of all treated cell lines. These results were unexpected because inhibition of CTLA-4 is traditionally associated with an increase in T lymphocyte proliferation. However, the activity of CTLA-4 in different cell types is incompletely understood. In line with our results, it was reported that treatment with Ipilimumab significantly reduced proliferation of CTLA-4-expressing CD4+ T-cells (71). Furthermore, the effect of CTLA-4 blockade seems to be associated with a decreased production of cytokines, such as IFN-γ, TNF-α, IL-13, and IL-17F, rather than with the induction of apoptosis (72). Although we did not evaluate apoptosis markers, differences in cell viability were not observed between cells incubated with Ipilimumab and untreated cells, suggesting that the reduction of proliferation might be associated with unknown signaling cascades or with extracellular signals, such as cytokines, that need to be studied to better understand the molecular mechanisms governing CTLA-4 functions in TNBC cells. Importantly, anti-proliferative effects of anti-CTLA therapies in tumor cells has novel prognostic and clinical implications.

We observed that the immune component was up-regulated in the CTLA-4 ERK1/2 activated-like subtype. GSEA screening clearly delineated enrichment of inflammation and immune infiltration signaling, as well as the antigen processing/presentation machinery in association to CTLA-4 activity. Interestingly, translocation of CTLA-4 to the T-cell membrane induces the inhibition of IL-2 production and secretion (72). The aberrant production of IL-2 has been documented in several tumors (73–76), including breast cancer (77). Accordingly, incubation with CD80 decreased the concentration of soluble IL-2 in HCC1937 cells, whereas treatment with Ipilimumab significantly increased soluble IL-2, which was able to sustain the proliferation of lymphocytes already expressing CTLA-4. It is known that CTLA-4 regulates transcription of IL-2 mainly by suppressing the activity of the PI3K/AKT cascade (78). Thus, we hypothesize that the reduction of secreted IL-2 might be due to either the inhibition of AKT-Thr308 phosphorylation or the over-activation of ERK/TGF-β axis, since it has been reported that TGF-β inhibits effector/memory T lymphoblast proliferation and IL-2 production (79, 80).

Our data suggest that Ipilimumab may stimulate CTLA-4-expressing tumor cells to secrete IL-2, contributing to the establishment of a favorable immunologic microenvironment, that may support the action of Ipilimumab. In our study, CTLA-4 expression and activation were correlated with higher immune infiltration of cells typically expressing CTLA-4 as an evasion mechanism (81–84), such as exhausted-like lymphoid cells, mainly CD8/CD4 T-cells and NK cells, as well as the myeloid compartment, including macrophages. Tumor microenvironment may influence immunotherapy responses by building complex interactions between tumor cells, immune cells, and secreted soluble factors. We described that CTLA-4 might regulate tumor immune microenvironment by enhancing relevant immunomodulators, such as IL-15 and CSF-1, which prime NK and M1 macrophages, two cells interconnected by their immunomodulatory functions. These data point out the existence of a fine crosstalk between immune infiltrated cells and CTLA-4 expressing tumor cells, that deserves further study.

At present, the possibility of including immune checkpoint inhibitors as part of primary TNBC treatment is being discussed (85–87). However, the lack of biomarkers for the selection of patients still limits the use of immune checkpoint blockade in TNBC therapy. Here we have found that, in addition to the expected expression of CTLA-4 on infiltrating lymphocytes, tumor cells from some patients, and cell lines, are also positive for the expression of CTLA-4 at the cell-surface. Interestingly, blockade of the receptor did not produce the same effect in all CTLA-4-expressing cell lines, and it seems to depend on the potential modulation of the canonical signaling cascades (AKT, ERK1/2) and the resulting production of regulatory cytokines, such as IL-2, suggesting that checkpoint inhibitors would work best in patients with tumors over-expressing CTLA-4 at the cell membrane. In fact, the immune signature score, which is well associated with response to anti-CTLA-4 treatment, revealed a high prevalence of predicted responders to immunotherapy in CLTA-4 activated patients in the analyzed datasets. Overall, these findings provide novel insights into the biological landscape of tumors over-expressing CTLA-4 and could lead to the identification of useful biomarkers for immunotherapy, increasing the spectrum of patients to be benefited.

An important limitation of this work is the small number of samples evaluated by immunochemistry, which prevented us from making any generalization of the results. To overcome this limitation, a larger number of tissue samples are currently being studied. Another restriction is the small number of cell lines analyzed that do not completely recapitulate TNBC heterogeneity. However, taking advantage of benchmark data we reinforced our analysis including 730 triple-negative tumors fulfilling the inclusion criteria. This allowed us to identify different biological patterns associated with CTLA-expression in tumors and in cell lines. We also defined relevant mechanisms and biological portraits of human tumors over-expressing activated CTLA-4, which may predict favorable responses to anti-CTLA-4 therapy.

In conclusion, we have found that CTLA-4 is expressed at the cell membrane of a subgroup of TNBC tumors and our results provide novel mechanistic data by which CTLA-4 regulates key signal transducers in tumor cells (Figure 6D). Moreover, blockade of CTLA-4 with Ipilimumab significantly activated molecular cascades that probably cooperate to create a favorable microenvironment that enhances immune responses against tumor cells (Figure 6D). These observations are promising since they suggest that CTLA-4 expressed at the tumor-cell surface might be itself a target of immune checkpoint inhibitors, and might also be a candidate biomarker for immunotherapy, which is urgently needed to provide robust tools to select patients who might benefit from this new therapeutic strategy.

## Data Availability Statement

Publicly available datasets were analyzed in this study. This data can be found here: the NCBI Gene Expression Omnibus (GSE25066, GSE2603, GSE19615, GSE21653, GSE76275, GSE102484, GSE76250, and GSE86948), the UCSC Xena Browser (https://xenabrowser.net/) (GDC TCGA Breast Cancer, BRCA), and cBioPortal (https://www.cbioportal.org/) (METABRIC).

## Ethics Statement

The studies involving human participants were reviewed and approved by American British Cowdray Medical Center (ABC Medical Center), Mexico City, Mexico. Written informed consent for participation was not required for this study in accordance with the national legislation and the institutional requirements.

## Author Contributions

LR-Z conceived the project and designed experiments, supervised laboratory staff, analyzed and discussed data, and wrote the manuscript. SR-C conceived and conducted all genomic data analyses, including preprocessing of raw microarrays or RNA-seq data, enrichment analysis and public gene signatures, analyzed and discussed data, and wrote the manuscript. MN-B performed cell proliferation and invasion assays, IL-2 ELISA, flow cytometry, and Western blot analysis. MC-B identified TNBC cases, created the patient database, performed immunohistochemistry, evaluated CTLA-4 staining, analyzed data, and wrote manuscript. JM-H supervised patient data revision, performed statistical analysis regarding CTLA-4 scores, and clinical features. CL-T supervised immunohistochemistry, performed pathological assessment, and CTLA-4 scores. RG-C and AZ-D coordinated study operation at the ABC Medical Center and participated in discussion and analysis of results. MI-S and JE-L supervised and performed Western blot analysis. JM and VC-M performed flow cytometry analysis. DO-P, DV-O, and ER-S participated in the selection of patients, retrieving of specimens, and reviewing of patient's data. PS-S and LT-U performed co-culture assays, analyzed data, and produced figures. All authors contributed to the article and approved the submitted version.

## Conflict of Interest

LR-Z has received personal fees from Bristol-Myers Squibb and Roche. The remaining authors declare that the research was conducted in the absence of any commercial of financial relationships that could be construed as a potential conflict of interest.
