# Supplementary figures and images for: Biological Landscape of Triple Negative Breast Cancers Expressing CTLA-4
Source: Front Oncol. 2020 Aug 5;10:1206. doi: 10.3389/fonc.2020.01206 (PMC7419680; doi:10.3389/fonc.2020.01206)

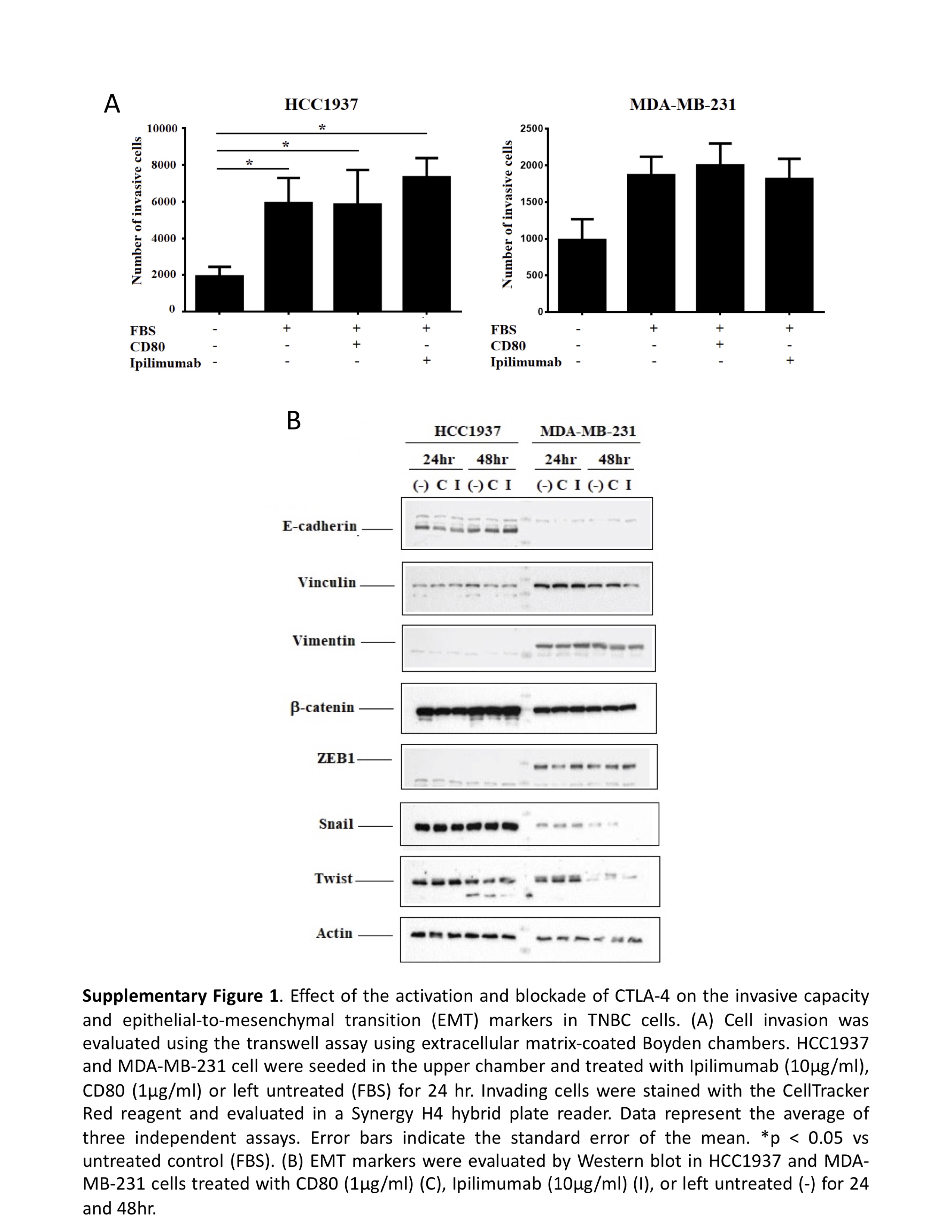

Supplement: Supplementary file 5 [file Image_1.JPEG]

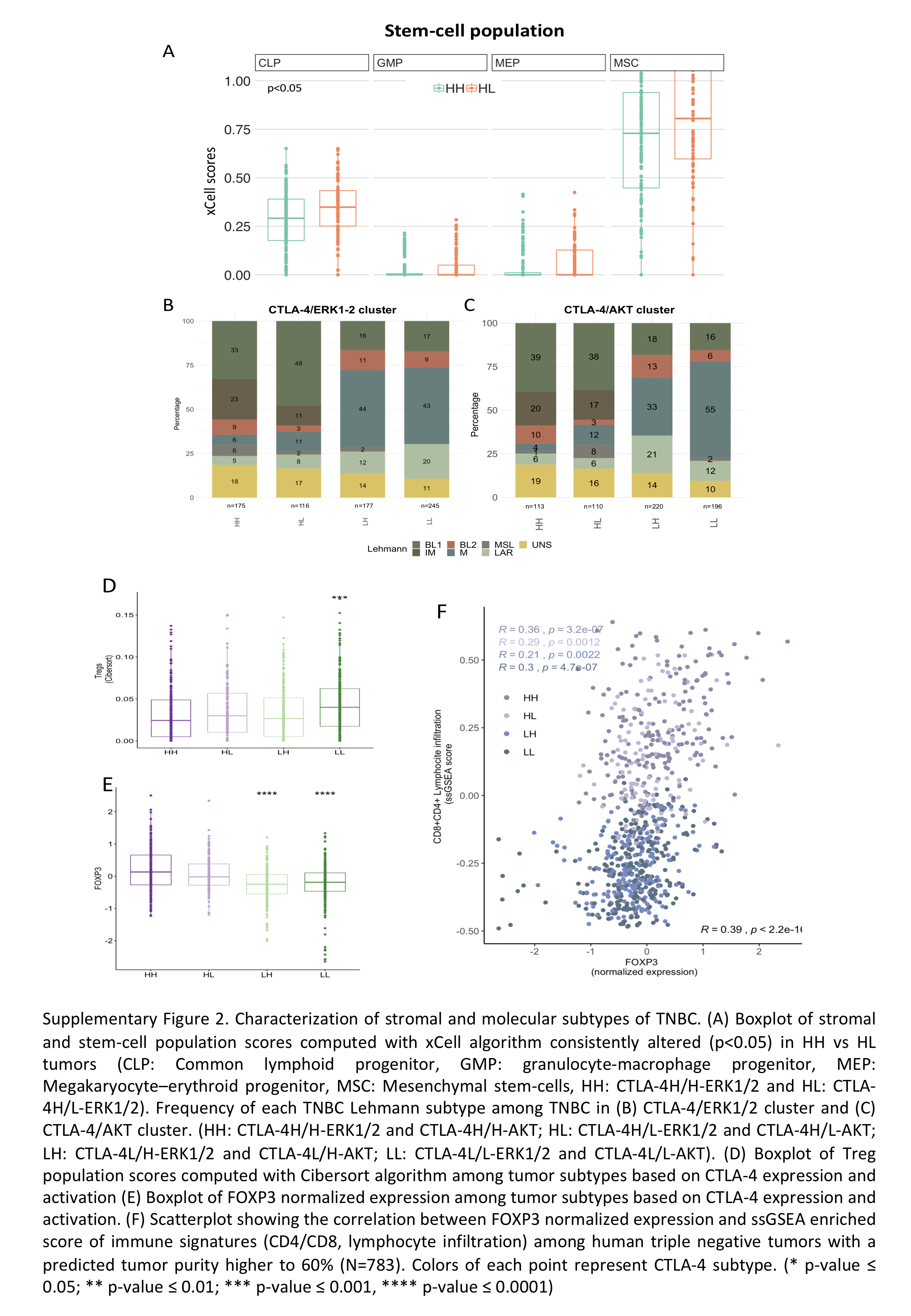

Supplement: Supplementary file 6 [file Image_2.JPEG]

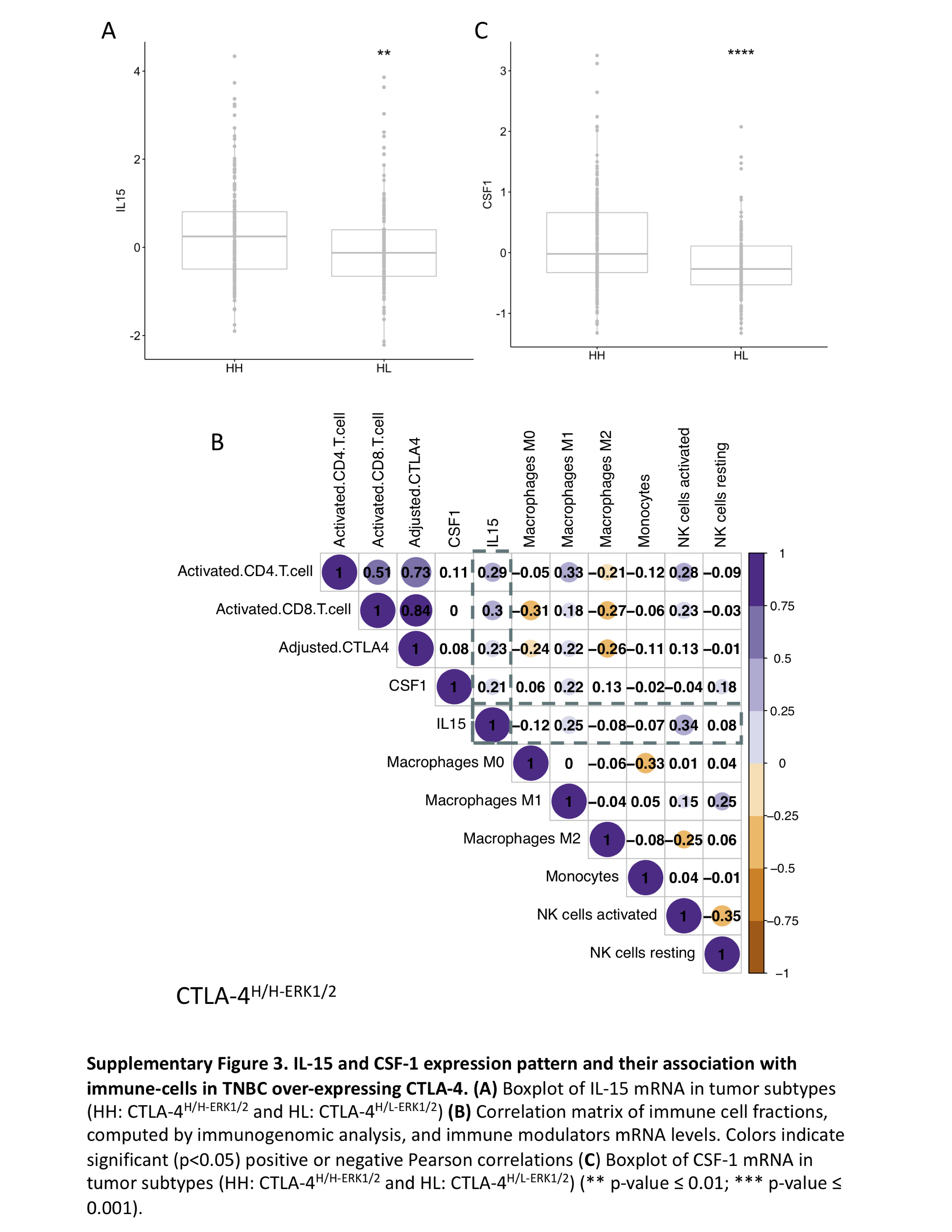

Supplement: Supplementary file 7 [file Image_3.JPEG]
